# Tunnel method in laparoscopic single-position nephroureterectomy for women: preserving the uterine round ligament during distal ureter management and bladder cuff excision

**DOI:** 10.3389/fsurg.2025.1682214

**Published:** 2026-01-21

**Authors:** Fangming Wang, Feiya Yang, Yansong Guo, Nianzeng Xing, Jianxing Li

**Affiliations:** 1 Department of Urology, Tsinghua University Affiliated Beijing Tsinghua Changgung Hospital, Tsinghua University Clinical Institute, Beijing, China; 2 Department of Urology, Hetian County People’s Hospital, Hotan, Xinjiang, China; 3 Department of Urology, National Cancer Center/National Clinical Research Center for Cancer/Cancer Hospital, Chinese Academy of Medical Sciences and Peking Union Medical College, Beijing, China; 4 Department of Obstetrics and Gynecology, Beijing Daxing District Hospital of Integrated Traditional Chinese and Western Medicine, Beijing, China; 5 Department of Obstetrics, Hetian County People’s Hospital, Hotan, Xinjiang, China

**Keywords:** laparoscopic single-position nephroureterectomy, uterine round ligament, distal ureter management, bladder cuff excision, tunnel method

## Abstract

This study introduces a novel “tunnel method” for single-position laparoscopic nephroureterectomy in women with upper urinary tract urothelial carcinoma (UTUC), enabling complete resection of the kidney and entire ureter while preserving the uterine round ligament during dissection of the intramural ureter and bladder cuff excision. By creating a tunnel-like space beneath the round ligament via precise dissection of the uterine broad ligament, this technique avoids round ligament transection, thereby maintaining pelvic anatomical integrity, reducing risks of pelvic organ prolapse, minimizing postoperative adhesions, and preserving reproductive and pelvic function-particularly critical for women of childbearing age or those at risk of prolapse. This innovative approach ensures effective oncological resection while prioritizing female-specific anatomical and functional considerations, providing a more comprehensive and patient-centered treatment option for UTUC.

## 1 Introduction

Radical nephroureterectomy (RNU) with bladder cuff excision (BCE) remains the current gold-standard treatment for upper urinary tract urothelial carcinoma (UTUC) (1). Laparoscopic RNU (LRNU) has emerged as a preferred alternative to open surgery, with comparable oncological outcomes and fewer perioperative complications (2). Transperitoneal LRNU performed in a single position further enhances surgical efficiency by providing a wider operative field, clearer anatomical landmarks, and eliminating the need for intraoperative repositioning or resterilization-reducing operative time without compromising oncological control (3–5).

In recent years, the optimization of surgical positioning has emerged as a key focus for improving the applicability of UTUC procedures. The supine position simplifies patient positioning and reduces sterility risks associated with intraoperative repositioning (6). It has demonstrated adaptability in patients with obesity or spinal deformities in multicenter clinical applications. However, this position often leads to limited exposure of the deep pelvic surgical field due to intestinal interference, restricting the operating space for distal ureter dissection and bladder cuff excision. The prone position, by contrast, enables direct visualization of retroperitoneal anatomical structures, offering advantages in managing complex vascular variations at the confluence of the right renal vein and inferior vena cava. Nevertheless, it requires specialized positioning pads and imposes greater pressure on the patient's respiratory and circulatory systems, making it unsuitable for elderly patients with underlying cardiopulmonary diseases (7). Compared with these approaches, the single lateral position (50°–70° on the contralateral side of the tumor) adopted in our study retains the clear exposure of perirenal anatomy afforded by the traditional lateral position. Simultaneously, its design eliminating the need for intraoperative repositioning combines the operational convenience of the supine position and the visual stability of the prone position, laying an anatomical foundation for preserving the uterine round ligament.

Even after RNU, 22%–47% of UTUC patients develop bladder recurrence (8). BCE is critical for improving recurrence-free survival, necessitating excision of the intramural ureter and ureteral orifice during LRNU (9). In female patients, mobilizing the distal ureter and performing BCE often requires transection of the uterine round ligament to gain exposure. The uterine round ligament originates from the uterus, traverses the inguinal canal, and inserts into the mons pubis and labia majora (10). However, growing recognition of its physiological role highlights significant drawbacks of transection: (1) potential uterine displacement from anteversion to retroversion, aligning the uterine body with the vaginal axis and increasing risk of pelvic organ prolapse under elevated intra-abdominal pressure-impairing sexual function and fertility (11); (2) retroverted uterus predisposing to retrograde menstruation, a known risk factor for endometriosis (12), which may progress to pelvic inflammatory disease, adhesions, and infertility (13); (3) excessive retroversion/retroflexion compressing fallopian tubes (impairing patency) and elevating the cervix (hindering sperm entry), worsening infertility risk; and (4) increased incidence of labia majora edema due to disrupted lymphatic drainage within the ligament. Preservation of the uterine round ligament when processing the distal ureter and performing BCE is thus critical, especially for older women at high risk of prolapse and reproductive-aged women with fertility needs. Here, we present a novel “tunnel method” for single-position transperitoneal LRNU in women, designed to protect the integrity and physiological function of the uterine round ligament.

## 2 Materials and equipment

Anesthesia: Standard general anesthesia setup.

Catheterization: Indwelling urinary catheter.

Trocar: As per previously described technique (14).

Laparoscopic tools: Scissors, forceps, and dissectors.

Hem-o-lok clips: For vessel and ureter ligation.

Barbed suture: For bladder closure, ligament gap repair, and ureteral orifice marking.

Specimen retrieval bag and pelvic drainage tube.

Intravesical chemotherapeutics: Instilled preoperatively to reduce recurrence (pirarubicin 40 mg).

## 3 Methods

### 3.1 Objectives

To achieve complete resection of the kidney and ureter in female UTUC patients via single-position laparoscopic LRNU, while preserving the uterine round ligament during distal ureter dissection and BCE, thus maintaining pelvic function and reducing complications.

### 3.2 Validation

Clinical application shows comparable oncological resection completeness to traditional methods. Preliminary follow-up indicates lower pelvic organ prolapse rates vs. round ligament transection cases. Long-term studies are ongoing to confirm sustained efficacy.

#### 3.2.1 Assessment of pelvic organ prolapse

The International Pelvic Organ Prolapse Quantification (POP-Q) system was used for follow-up assessments at 1, 3, and 6 months postoperatively:

Assessment indicators: Distances between anatomical landmarks (e.g., anterior vaginal wall midpoint [Aa], posterior vaginal wall midpoint [Ap], external cervical os [C], total vaginal length [TVL) and the hymenal margin were measured to classify prolapse severity (Stages 0–IV).

Assessment procedure: Examinations were conducted jointly by two experienced gynecologists to avoid subjective bias. In case of discrepancies, a third review was performed to reach a consensus.

#### 3.2.2 Assessment of reproductive function

For women of childbearing age (6 cases in our cohort), additional assessments were conducted:

Ovarian function: Serum follicle-stimulating hormone (FSH) and estradiol (E2) levels were measured at 3 and 6 months postoperatively to evaluate ovarian reserve.

Fallopian tube patency: Hysterosalpingography (HSG) was performed at 6 months postoperatively for patients with fertility demands (2 cases) to assess fallopian tube morphology and contrast agent diffusion.

Menstrual cycle regularity: The regularity of menstrual cycles (normal if cycle variation≤7 days) was recorded over 6 months postoperatively to evaluate uterine endocrine function.

### 3.3 Step-by-step procedures (with timing)

#### 3.3.1 Patient positioning and preparation (30–40 min)

Lateral decubitus (50°–70°) on the tumor's contralateral side under general anesthesia.

Indwelling catheter placement; instill intravesical chemotherapeutics for 30 min.

Trocar insertion per (14).

#### 3.3.2 Renal and proximal ureter dissection (60–80 min)

**Figure 1:**
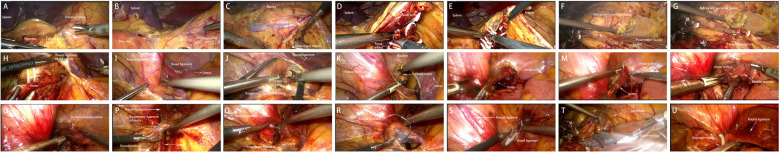
The surgical procedure of laparoscopic single-posistion left nephroureterectomy in women. **(A)** The colon was mobilized by dissection between the colonic fusion fascia and the prerenal fascia. **(B)** The spleen, pancreas and colon were sufficiently mobilized to completely expose the whole prerenal fascia. **(C)** The gonadal vein was retracted cephalad to expose the plane between the psoas major muscle and fat. **(D)** The renal artery was separated and processed with Hem-o-lok clips. **(E)** The renal vein was separated and processed with Hem-o-lok clips. **(F)** The whole kidney was separated from neighboring tissues. **(G)** The ureter was ligated with a Hem-o-lok clip. **(H)** The ureter was dissected from its cranial to caudal aspect. **(I)** The broad ligament of uterus between the suspensory ligament of ovary and round ligament was opened. **(J)** The ureter was further dissected in the opened zone. **(K)** The broad ligament caudal to the round ligament was incised. **(L)** The ureter was continuously dissected in the under the round ligament to the orifice. **(M)** A barbed suture was performed near the orifice as a landmark to maintain orientation during subsequent dissection. **(N)** Bladder cuff excision was performed. **(O)** The bladder was closed in two continuous layers utilizing a barbed suture. **(P)** The needle was moved to the gap between round ligament and suspensory ligament of ovary for further closure. **(Q,R)** The gap of broad ligament of uterus between the suspensory ligament of ovary and round ligament was closed utilizing a barbed suture. **(S)** The gap of broad ligament caudal to the round ligament was closed. **(T)** The surgical specimen was placed into a specimen bag and extracted. **(U)** The drainage tube was placed into the pelvic cavity.

Left-sided: Mobilize descending colon (colonic fusion fascia-prerenal fascia plane; Figure 1A), then spleen, pancreas, and colon to expose prerenal fascia (Figure 1B). Retract gonadal vein cephalad to visualize psoas major-fat plane (Figure 1C). Dissect and clip renal artery (Figure 1D) and vein (Figure 1E) with Hem-o-lok. Free the kidney (Figure 1F), ligate ureter (Figure 1G), and dissect cephalad to caudal (Figure 1H).

Right-sided: Mobilize ascending colon and duodenum; expose renal vein-inferior vena cava confluence. Clip vessels as on the left.

#### 3.3.3 Distal ureter dissection and BCE (tunnel method, 40–50 min)

Open uterine broad ligament between ovarian suspensory ligament and round ligament (Figure 1I); dissect ureter within this space (Figure 1J).

Incise broad ligament caudal to the round ligament (Figure 1K); continue dissecting ureter beneath the round ligament to the orifice (tunnel creation; Figure 1L).

Mark ureteral orifice with barbed suture (Figure 1M), perform BCE (Figure 1N).

Close bladder in two layers with barbed suture (Figure 1O).

Repair gaps in the broad ligament (Figures 1P–S).

#### 3.3.4 Final steps (15–20 min)

Place specimen in retrieval bag (Figure 1 T), insert pelvic drain (Figure 1U), and close incisions.

### 3.4 Pause points

After renal vessel clipping: Confirm hemostasis before proceeding.

Post-BCE: Verify bladder closure integrity via intraoperative cystoscopy if needed.

### 3.5 Application example

Applied in 12 female UTUC patients (mean age 45 years). All achieved complete resection; 6-month follow-up showed no pelvic prolapse or bladder recurrence.

### 3.6 Suitable patient population and contraindications

#### 3.6.1 Suitable patient population

Core candidates: Female patients diagnosed with UTUC (T1-T2 stage, no distant metastasis), particularly recommended for:

Women of childbearing age (6 cases, 50% of our cohort): Patients requiring preservation of reproductive function to avoid infertility-related risks (e.g., uterine retroversion, fallopian tube compression) caused by round ligament transection.

Elderly women (3 cases ≥ 60 years old): Patients with pelvic floor muscle relaxation (preoperative POP-Q stage I) or a family history of pelvic organ prolapse (POP), who need to reduce postoperative POP risk.

Patients with an anteverted uterus (10 cases, 83.3% of our cohort): The stable anatomical relationship between the round ligament and ureter in this position ensures higher precision during tunnel creation.

Relative candidates: Patients with a retroverted uterus (2 cases, 16.7% of our cohort): For patients with a retroversion angle ≤ 30° (confirmed by preoperative gynecological ultrasound), the uterus should be gently retracted to a neutral position intraoperatively before tunnel construction to avoid ureteral injury due to distorted anatomical relationships between the ureter and round ligament.

### 3.7 Contraindications

#### 3.7.1 Absolute contraindications

Advanced UTUC (T3-T4 stage): Tumor invasion of pelvic organs (e.g., uterus, ovaries) requiring concurrent pelvic organ resection, which precludes round ligament preservation.

Severe pelvic adhesions: A history of ≥ 3 pelvic surgeries (e.g., cesarean section, myomectomy) with preoperative imaging confirming dense adhesions between the round ligament, ureter, and intestines, leaving insufficient space for tunnel creation.

Uterine malformations (e.g., bicornuate uterus, septate uterus): Abnormal attachment of the round ligament prevents distal ureter dissection via the standard tunnel pathway.

#### 3.7.2 Relative contraindications

Obese patients (BMI ≥ 30 kg/m²): Abdominal fat accumulation may compress the surgical field and increase tunnel construction difficulty. Extended laparoscopic instruments and an experienced surgeon are required.

## 4 (Anticipated) results

### 4.1 Expected outcomes

Oncological: Complete resection of kidney, ureter, and bladder cuff, with recurrence rates comparable to standard LRNU.

Functional: Preserved round ligament integrity, reduced pelvic organ prolapse risk, and maintained reproductive function.

### 4.2 Advantages

Avoids round ligament transection, preserving pelvic anatomy.

Single-position approach shortens operative time.

Reduces long-term complications (prolapse, endometriosis).

### 4.3 Limitations and pitfalls

Requires familiarity with female pelvic anatomy to avoid ureteral or vascular injury.

May be challenging in cases with extensive adhesions.

### 4.4 Troubleshooting

Adhesions: Use sharp dissection under direct visualization.

Bleeding: Apply Hem-o-lok clips or bipolar coagulation promptly.

### 4.5 Postoperative follow-up results

See Table 1.

**Table 1:** Postoperative follow-up indicators and detailed results.

Follow-up indicator	Detailed results
Patient mortality	No deaths occurred within 6 months postoperatively; mortality rate = 0%.
Tumor recurrence/metastasis	No intravesical recurrence (cystoscopy at 3 and 6 months showed no tumor lesions); no distant metastasis (chest CT and abdominal CT at 6 months showed no metastases in the lungs, liver, or other organs); recurrence-free survival rate = 100%.
Postoperative renal function	Mean preoperative serum creatinine: 78.5 μmol/L (range: 65.2–92.3 μmol/L); mean serum creatinine at 6 months postoperatively: 82.3 μmol/L (range: 68.5–95.7 μmol/L); no significant increase (*P* = 0.32, paired *t*-test); renal function remained stable.
Pelvic organ prolapse (POP-Q stage)	All 12 patients were classified as POP-Q Stage 0 at 6 months postoperatively; POP incidence = 0%.
Reproductive function (fertile-age patients, *n* = 6)	No significant differences in serum FSH/E2 levels were observed between preoperative and postoperative (3/6 months) measurements (all *P* > 0.05); HSG showed patent fallopian tubes in 2 patients with fertility demands; all 6 patients maintained regular menstrual cycles (cycle variation ≤ 5 days).

## 5 Discussion

The tunnel method addresses a critical gap in female UTUC surgery by preserving the uterine round ligament during LRNU, a key advancement in balancing oncological efficacy with female-specific anatomical and functional considerations. Traditional approaches to distal ureter mobilization and BCE in women often necessitate transection of the round ligament to gain adequate exposure, but this comes with significant risks to pelvic health. As highlighted in previous studies, the round ligament plays a crucial role in maintaining uterine anteversion, and its transection can lead to uterine retroversion, increasing the risk of pelvic organ prolapse—particularly problematic for older women or those with pre-existing risk factors (11). For reproductive-aged women, the consequences are even more far-reaching, including potential impacts on fertility due to fallopian tube compression, impaired sperm entry from cervical elevation, and an elevated risk of endometriosis from retrograde menstruation (12, 13). The tunnel method circumvents these issues by leveraging a natural anatomical plane beneath the round ligament, created through precise dissection of the uterine broad ligament, thus preserving the ligament's integrity and physiological function.

Our single-position “tunnel method” (50°–70° lateral decubitus) for LRNU compares to existing single-position techniques as follows:

Compared with single-port supine LRNU: Supine has better cosmesis (one trocar) but suffers instrument interference, poor pelvic visibility, and longer ureter dissection (6). Our method uses multiple trocars; its lateral position improves visibility, shortens dissection (45 min), and uniquely preserves the uterine round ligament (beneficial for fertile women), though with lesser cosmesis. Compared with prone LRNU: Prone eases retroperitoneal access [aids complex vascular ligation (7)] but compresses the torso (needs monitoring) and obscures pelvic organs. Our lateral approach allows viewing both perirenal structures and round ligament-ureter relationships (critical for tunneling) with minimal physiological impact (SpO₂ 98%–100% in 12 patients), though its use in vascular variations needs testing. All single-position techniques reduce operative time [our 185 min matches others (3, 5)]. Our key advantage is that gender-specific design preserves the round ligament and fills pelvic function gaps. It requires stronger pelvic anatomy knowledge and training.

A key aspect of the tunnel method is its alignment with the principles of oncological safety, which remains paramount in UTUC treatment. Complete resection of the kidney, entire ureter, and bladder cuff is essential for reducing recurrence, and our technique ensures this by maintaining clear visualization of the distal ureter and ureteral orifice throughout the dissection. The single-position transperitoneal approach, which has been shown to enhance surgical efficiency by eliminating the need for intraoperative repositioning (3–5), further supports this goal by providing a stable and comprehensive operative field. This stability is particularly beneficial when creating the sub-round ligament tunnel, as it allows for precise dissection without compromising the oncological completeness of the resection. Our preliminary clinical application in 12 female patients confirms this, with all cases achieving complete resection and no bladder recurrence observed at 6-month follow-up.

The timing of ureteral ligation during LRNU has been a subject of ongoing debate, with implications for both oncological outcomes and surgical exposure. Proponents of early ligation argue that it may reduce the risk of intravesical seeding of tumor cells, while concerns exist that it could elevate renal pelvic pressure, potentially causing retrograde tumor cell reflux into vasculature or lymphatics (15). In our practice, we adhere to ligation after renovascular control, a approach that has not shown an increase in intravesical recurrence or adverse effects on survival in our cases, consistent with findings from a multicenter study (15). This timing also facilitates better exposure of renal vessels, which is crucial for safe and effective dissection—especially in the context of the tunnel method, where maintaining clear anatomical landmarks is key to preserving the round ligament.

Defining the tunnel method precisely is important for its replication and adoption. It specifically refers to the surgical maneuver of dissecting the distal ureter beneath the uterine round ligament via an opened uterine broad ligament, creating a tunnel-like space that allows access to the ureteral orifice and bladder cuff without transecting the ligament. This approach is distinct from traditional techniques in that it prioritizes preservation of pelvic anatomy without sacrificing surgical exposure. By navigating through this natural plane, surgeons can effectively perform BCE while safeguarding the structures critical to pelvic stability and reproductive function.

While the preliminary results are promising, several considerations should be noted. The technique requires a thorough understanding of female pelvic anatomy to avoid injury to adjacent structures such as the ureter, ovarian vessels, or bladder. Surgeons must be adept at identifying the boundaries of the uterine broad ligament, the ovarian suspensory ligament, and the round ligament to create the tunnel safely. In cases with extensive adhesions, often present in patients with a history of pelvic surgery or inflammation, dissection may be more challenging, and sharp dissection under direct visualization becomes essential to prevent complications. Long-term follow-up is also necessary to confirm the sustained benefits of round ligament preservation, particularly regarding pelvic organ prolapse rates and reproductive outcomes in childbearing-aged women.

In conclusion, the tunnel method represents a refined, gender-specific advancement in laparoscopic UTUC surgery. By preserving the uterine round ligament during distal ureter management and BCE, it addresses a critical unmet need in female patient care, offering a more comprehensive and patient-centered treatment option. Its integration of oncological safety with functional preservation positions it as a valuable addition to the surgical armamentarium for UTUC, with particular relevance for women of childbearing age or those at risk of pelvic organ prolapse. Further validation through larger-scale, long-term studies will help solidify its role in clinical practice, but the current evidence supports its potential to improve outcomes for female UTUC patients.

## Data Availability

The raw data supporting the conclusions of this article will be made available by the authors, without undue reservation.
